# Long-Range Dispersal and High-Latitude Environments Influence the Population Structure of a “Stress-Tolerant” Dinoflagellate Endosymbiont

**DOI:** 10.1371/journal.pone.0079208

**Published:** 2013-11-05

**Authors:** D. Tye Pettay, Todd C. LaJeunesse

**Affiliations:** 1 Department of Biology, Pennsylvania State University, University Park, Pennsylvania, United States of America; 2 College of Earth, Ocean, and Environment, University of Delaware, Lewes, Delaware, United States of America; King Abdullah University of Science and Technology, Saudi Arabia

## Abstract

The migration and dispersal of stress-tolerant symbiotic dinoflagellates (genus *Symbiodinium*) may influence the response of symbiotic reef-building corals to a warming climate. We analyzed the genetic structure of the stress-tolerant endosymbiont, *Symbiodinium glynni* *nomen nudum* (ITS2 - *D1*), obtained from *Pocillopora* colonies that dominate eastern Pacific coral communities. Eleven microsatellite loci identified genotypically diverse populations with minimal genetic subdivision throughout the Eastern Tropical Pacific, encompassing 1000’s of square kilometers from mainland Mexico to the Galapagos Islands. The lack of population differentiation over these distances corresponds with extensive regional host connectivity and indicates that *Pocillopora* larvae, which maternally inherit their symbionts, aid in the dispersal of this symbiont. In contrast to its host, however, subtropical populations of *S. glynni* in the Gulf of California (Sea of Cortez) were strongly differentiated from populations in tropical eastern Pacific. Selection pressures related to large seasonal fluctuations in temperature and irradiance likely explain this abrupt genetic discontinuity. We infer that *S. glynni* genotypes harbored by host larvae arriving from more southern locations are rapidly replaced by genotypes adapted to more temperate environments. The strong population structure of *S. glynni* corresponds with fluctuating environmental conditions and suggests that these genetically diverse populations have the potential to evolve rapidly to changing environments and reveals the importance of environmental extremes in driving microbial eukaryote (e.g., plankton) speciation in marine ecosystems.

## Introduction

Marine microrganisms have the potential to disperse great distances and may therefore show little population differentiation over large geographic regions [1,2]. The genetic connectivity of these organisms is likely influenced by numerous factors including changes in water quality, nutrients, irradiance and temperature as well as prevailing ocean currents [2]. While some species appear geographically widespread, phylogenetic and emerging population genetic data are indicating that many widespread taxa actually comprise numerous cryptic species displaying limited geographic ranges and/or populations of cosmopolitan species are not panmictic [3–8]. Despite some progress, the microevolution of eukaryotic microalgae remains poorly understood because culturing is often required prior to genetic analyses [3,8], which can be laborious and highly selective [9]. In contrast, widespread endosymbiotic dinoflagellates in the genus *Symbiodinium* are ideal models for population genetic studies since numerous samples may be acquired from various marine invertebrate hosts and habitats over a range of spatial and temporal scales [10–13]. 

Reef coral communities in the eastern Pacific prosper under sea surface temperatures (SSTs), nutrient concentrations and irradiance levels that fluctuate widely between seasons [14]. These environmental extremes and isolation from the central Pacific explain the depauperate community assemblage of reef corals that harbor *Symbiodinium* [14]. The most distinctive feature of these communities is that branching *Pocillopora* can represent 90-100% of the live coral cover in near shore hard bottom habitats and encompass a latitudinal range of both tropical (Eastern Tropical Pacific or ETP) and subtropical (Gulf of California or GoC) regions. While approximately eight or nine morphospecies of *Pocillopora* have been reported from the eastern Pacific [14], recent genetic and ecological data question the validity of this diversity [15,16]. According to nuclear and mitochondrial sequence variation, as well as differences in allelic variability among microsatellite loci, it appears that most of the region’s *Pocillopora* may actually comprise a single genetically definable species designated as type 1 [16]. Populations of this coral (type 1) exhibited little discernable differentiation across the entire eastern Pacific despite habitat patchiness, strong environmental gradients and complex surface currents that may interrupt dispersal [14,16,17].

The ecological success of type 1 *Pocillopora* throughout the eastern Pacific may in part be attributed to their associations with the intracellular symbiont, *Symbiodinium D1* [18]. *Symbiodinium D1* is defined genetically by internal transcribed spacer (ITS) region 2 sequence identity and is referred to here as *S. glynni* *nomen nudum* (*sensu* [18]). *Symbiodinium glynni* appears ecologically specialized to the *Pocillopora* in this region [19,20]. This symbiont is extremely common among type 1 *Pocillopora* and dominates the majority of colonies in near shore locations, especially along the coastal mainland of central and southern Mexico where it appears to be the only symbiont present in populations of this animal [18]. Like many other symbioses involving species in clade D, colonies harboring *S. glynni* possess a greater thermal tolerance and are more likely to survive when exposed to stressful temperatures [18,21]. As a consequence of its abundance and thermal tolerance, the symbiosis between *Pocillopora* spp. and *S. glynni* is vital to the present and future stability of shallow water communities of the region. 

Stress tolerant *Symbiodinium* offer one mechanism by which some reef corals cope with warm or environmentally unstable conditions [20,22,23]. However, geographic separation may prevent coral populations exposed to increasing stress from developing symbioses with better-adapted symbionts from other regions. Population genetic data can reveal patterns of genetic connectivity, allowing inferences to be made on the ranges of, and barriers to, dispersal and providing a better understanding of how certain symbiont species may spread as environmental conditions change. Genetic and ecological data indicate that clade D is comprised of numerous operational taxonomic units, or species [12,20,24], yet nothing is known of their population structure. Populations of some *Symbiodinium* spp. separated by small distances exhibit significant differences in microsatellite allele diversity and suggest that their effective dispersal is limited to 5 or 10 kilometers ([10,25–28]; but see [29]). However, population genetic studies of *Symbiodinium* remain few and substantially more data are needed to evaluate the generality of genetic structuring in a species’ metapopulation. 

The abundant and widespread populations of eastern Pacific *Pocillopora* where used to study the genetic diversity and connectivity of its “stress-tolerant” symbiont. We hypothesized that the lack of detectable genetic differentiation among *Pocillopora* type 1 populations across both tropical and subtropical regions of the eastern Pacific [16] should impart similar patterns on populations of its co-migrating symbiont, which is transmitted vertically from generation to generation [30,31]. Samples of *Pocillopora* with *S. glynni* were collected from populations located in several biogeographic provinces and spanning over 3,400 km from the Galapagos Islands at the equator to the southern Gulf of California (Sea of Cortez) at approximately 24°N. In the first large-scale population genetic examination of a Clade D *Symbiodinium* spp., eleven microsatellite loci were analyzed and the population genetic structure compared with previously published data on the host.

## Materials and Methods

### Sample collections

Samples analyzed in this study were part of a larger and previously published biogeographical analysis of *Pocillopora* spp. symbionts across the eastern Pacific [18,19]. Fragments (~2 cm^2^) of *Pocillopora* colonies were collected by SCUBA from six sampling locations and preserved in either a high salt, 20% DMSO buffer [32] or 95% ethanol and stored at -20 °C until DNA extraction. At each regional location, samples were collected along linear transects at depths ranging from two to ten meters at each of one to four sites separated by as much as 50 km. Morphologically distinct colonies were sampled at least 3 meters apart to avoid sampling clonemates resulting from fragmentation. Sample collection dates for each location are as follows: Gulf of California (GoC) in May 2006 and June 2008, Banderas Bay (BB) and Clipperton Atoll (CLP) in April 2007, Gulf of Tehuantepec (OAX) in May 2008, Gulf of Panama (PAN) in January 2009, and the Galapagos Islands (GAL) in March 2007. Mexico, issued by the Mexican Federal Government through CONAPESCA, and Clipperton Atoll samples were acquired under collecting permits issued to the Universidad Autonoma de Baja California Sur and the Universidad de Guadalajara, while Panama samples were collected through the Smithsonian Tropical Research Institute (permit # SC/A-1-09) and the Galapagos Islands samples were collected through Charles Darwin Research Station and the Galápagos National Park Service. This work was conducted in accordance with IACUC guidelines at Penn State University. A total of 402 samples representing five different *Pocillopora* morphospecies were analyzed (Table 1). While samples included different morphospecies of host, genetic analysis shows them all to belong to *Pocillopora* type 1.

**Table 1:** Summary statistics by location including number of colonies sampled, number of unique MLGs of *S. glynni*, clonal richness (R), haploid diversity (h), information index (I), and probability of identity (PI).

**location**	**number of samples**	**unique MLGs**	**clonal richness (R)**	**haploid diversity (h)**	**information index (I)**	**private alleles**	**probability of identity (PI)**
Gulf of California (GoC)	137	64	0.46	0.43 (0.09)	0.85 (0.20)	12	1.87E-06
Banderas Bay (BB)	155	87	0.56	0.40 (0.10)	0.92 (0.26)	13	7.76E-07
Gulf of Tehuantepec (OAX)	71	40	0.56	0.44 (0.08)	0.87 (0.18)	1	1.97E-06
Clipperton Atoll (CLIP)	7	6	0.83	0.33 (0.10)	0.58 (0.19)	1	6.49E-05
Gulf of Panama (PAN)	21	7	0.30	0.30 (0.09)	0.46 (0.14)	0	7.55E-04
Galapagos Islands (GAL)	11	11	1.00	0.44 (0.11)	0.88 (0.23)	2	3.44E-07
Total	402	215	0.53	0.39 (0.04)	0.76 (0.08)		4.09E-09

Numbers in parenthesis represent the standard error for a particular statistic.

### Molecular-genetic identification

Nucleic acid extractions were conducted and the dominant resident symbiont was identified by denaturing gradient gel electrophoresis (DGGE) fingerprinting of the partial 5.8S and internal transcribed spacer (ITS) region 2 [32]. Samples where *S. glynni* (*D1*) was detected were utilized for this study. 

### Microsatellite analysis

A subset of samples was initially screened with fifteen microsatellite loci developed for Clade D *Symbiodinium* to determine the degree of allelic polymorphism for these populations [12,24]. One primer set, D1Sym77, amplified two distinct loci and each was scored separately (see Text S1). Eleven out of fifteen loci were sufficiently polymorphic and used to determine the population structure of *Symbiodinium glynni* in the eastern Pacific (Table S1). GenePop (version 4.0.11, [33]) was used to examine the degree of linkage among these loci in pairwise comparisons. 

Fluorescently labeled fragments were analyzed at the Pennsylvania State University Genomics Core Facility according the methods of Pettay & LaJeunesse [11,12] and Wham et al. [24]. Microsatellites are known to produce stutter due to polyermase slippage during PCR, so only the dominant peaks were scored. Multilocus genotypes (MLGs) were constructed from fragment size data gathered for each sample and occasionally reanalyzed to confirm the existence of mixed genotypes and unusual or rare fragment sizes. Recovering multiple alleles at a locus (or loci) indicates the presence of two or more MLGs of symbiont within a single sample. In cases where mixtures were found, only the dominant fragment at each locus (i.e., highest peak) was used to construct a single MLG for that sample (see Text S1). While it has been shown that preferential amplification of certain fragments, usually the smallest, can occur with microsatellites, experimental mixtures using these loci have been shown to correctly amplify the fragment in highest concentration (data not shown). Therefore, it is believed that constructing MLGs using the dominant fragments adds little bias, yet allows a maximum number of MLGs to be recovered (see Text S1). Lastly, the asexual reproduction of clonal organisms can negatively bias statistical calculations based on allele frequencies (see Text S1) [34], so all analyses (unless stated otherwise) were conducted with duplicated MLGs removed at each sampling location. 

### Data analysis

The software Structure (Version 2.3.2) was used to overcome biases of assigning populations by location by using the microsatellite data to cluster MLGs based on their genetic similarities irrespective of sample origin. The validity of using Structure with haploid genomes is discussed in the Text S1. Structure analyses to investigate differentiation across the eastern Pacific were conducted using an admixture model with correlated allele frequencies and were run from K = 1 to 10 with five runs per K and a burnin of 100,000 and 1,000,000 reps after the burnin. A plot of the log probability of the data for a given K (ln P(D)) versus K was derived from the structure results, along with an analysis of the second order rate of change of K following the method by Evanno et al. [36] and implemented using Structure Harvester (v0.56.4) by Earl [37] to determine the appropriate clustering of individuals. Five runs per K were utilized to verifying consistency between runs, with the run having the highest ln P(D) for the appropriate K used to construct Structure plots and inform clustering. Graphic displays of Structure plots were manipulated (i.e., color and sample order) using DISTRUCT [38]. Additional analyses were performed on the ETP region only with the additional use of the location prior feature, which performs better in the presence of weak population structuring ([39]; see results). To graphically compare population clustering for host and symbiont, Structure analyses were also conducted on previously published population data for the host, *Pocillopora* type 1 [16], using a correlated allele model with admixture and location prior and were run for K = 2 with five runs per K and a burnin of 100,000 and 1,000,000 reps after the burnin. Additional Bayesian analyses were implemented using the program BAPS (Version 5.3) to confirm Structure clustering and is described in Text S1 [40].

To investigate the possibility of population differentiation according to *Pocillopora* morphospecies, Structure analyses were conducted on data from the three locations where sampling was adequate to conduct such a comparison; GoC, BB and OAX. For these analyses, data were grouped according to host morphology with identical MLGs removed within each morphological grouping. The analytical parameters used were identical to those for geography, with the exception that the location prior setting was used to better assist with weak structuring [39] and the maximum K was set to eight. 

Analyses of molecular variance (AMOVA) were then conducted on geographic populations with regional structuring as defined by Structure and by morphospecies to determine the degree of differentiation. AMOVA’s along with a permutation procedure were performed in GenAlEx [35] to test for significant difference in genetic diversity between populations [41]. The AMOVA produces variance components along with Φ-statistics (F-statistic analogs), which partition genetic variation at different hierarchical levels [41]. The significance of the variance components and Φ-statistics were then tested using 10000 permutations and a Bonferroni corrected α = 0.05. In addition to quantifying genetic differentiation, GenAlEx was used to calculate several summary statistics for each Structure population and/or sampling location, including haploid genetic diversity (h), information index (I), clonal richness (R) and the probability of identity (PI) (see Text S1 for descriptions). Since PI values may be affected by population substructure [42], it was calculated for each location and for the entire dataset.

### Environmental data

Monthly averages for sea surface temperatures (SSTs; °C) and photosynthetically active radiation (PAR; Einsteins m^-2^ day^-1^) were calculated for each sampling location using data for years 2000 to 2009 obtained from the Giovanni online data system, developed and maintained by NASA Goddard Earth Sciences (GES) Data and Information System (DISC). Approximately 24 km^2^ around each sampling location was selected and data retrieved from the SeaWiFS (PAR) and MODIS-Aqua (SST) databases. Scatter plots were drawn using monthly averages and standard deviations. To compare the variation, box plots were drawn using annual means, first and third quartiles, and extreme maximum and minimum annual values. 

## Results

### ITS2-DGGE fingerprinting and sequencing

Each of the MLGs characterized in this study possessed a single ITS2 sequence (D1) that dominated their ribosomal array [20,43,44], contrasting with most other Clade D types characterized by this method. Additionally, D1 is the only Clade D *Symbiodinium* known to occur in the eastern Pacific and to associate with corals in the genus *Pocillopora* at abundances that are physiologically relevant [18,19]. These genetic and ecological distinctions indicated that *S. glynni* *nomen nudum* represents a distinct operational taxonomic unit (*sensu* [20]), however, it still awaits formal taxonomical description.

### Microsatellite data

The number of alleles per locus ranged from 3 to 29, however, the effective number of alleles (A_e_) per locus ranged from 1.02 to 6.22 (Table S1). Allele frequencies for each location ranged from 0.011 to 1.00 (Table S2). The frequencies of putative null alleles were rare among loci ranging from 0 for loci D1Sym88 and D1Sym92 to a value of 0.079 for D1Sym11. 

Numerous alleles were private to a particular sampling location or region (Table S2). The GoC possessed 12 alleles involving five loci (D1Sym14, 17, 34, 77b & 88) that were unique to the region. Banderas Bay possessed 13 alleles from three loci (D1Sym9, 34 & 77b), GAL possessed 2 alleles from one locus (D1Sym34), and CLP and OAX each possessed 1 allele at one locus (D1Sym34 & D1Sym14) found only in these locations. All alleles in PAN were found in at least one other location. 

Two hundred and fifteen different genotypes were scored among four hundred and two MLGs obtained. Seventy-five, or 18.7 percent, of samples possessed multiple MLGs (i.e. more than one allele at one or more loci). The majority of these samples (n = 51) possessed two alleles at just one locus. Another 24 samples contained two alleles at multiple loci (11, 5, 2, and 6 samples possessed allele variation at 2, 3, 4 and 5 loci, respectively). Multiple alleles were never observed for six or more loci or for the conserved loci D1Sym88 and D1Sym92. 

The probability of identity (P_(ID)_) that two samples with the same MLGs may not have originated from the same clone lineage was exceedingly low for most locations and ranged from 7.55 x 10^-4^ to 7.76 x 10^-7^ (Table 1). All of these values, except one, were below the 0.0001 criteria recommended by Waits et al. [42]. The overall P_(ID)_ based on data from all locations was 4.09 x 10^-9^. Therefore, there was a 1 in two hundred and fifty million probability that identical genotypes from separate locations are of independent origins. 

Clonal richness (R) or genotypic richness ranged from 0.30 to 1.00 for each location (Table 1). Values approaching 0 indicate high clonality whereas values of 1.00 are indicative of populations where no repeated genotypes were sampled. Haploid diversity indices ranged from 0.30 to 0.44 with an overall average of 0.39 for all locations, while the information index ranged from 0.92 to 0.46 with an average of 0.76 (Table 1). The mean genotypic richness for all locations was 0.51 indicating that a large proportion (~ 49%) of MLGs were detected two or more times. Some “locally prevalent” clones were unusually common within and among sites in all sampling locations except GAL and CLP. In one case a MLG was recovered from nineteen different colonies.

A pairwise analysis of linkage among loci revealed that ~93% of the pairwise comparisons were unlinked (Bonferroni corrected; α = 0.05), which strongly suggests frequent sexual recombination among *S. glynni* MLGs [10]. In general, linkage between paired loci changed based on the population under analysis, with no pair of loci linked in all locations. The linkage for some loci is believed to be a consequence of the clonal evolution of these dinoflagellates or, in some cases, evidence of weak differentiation over small spatial scales within and between sites from a particular location.

### Population differentiation

**Figure 1:**
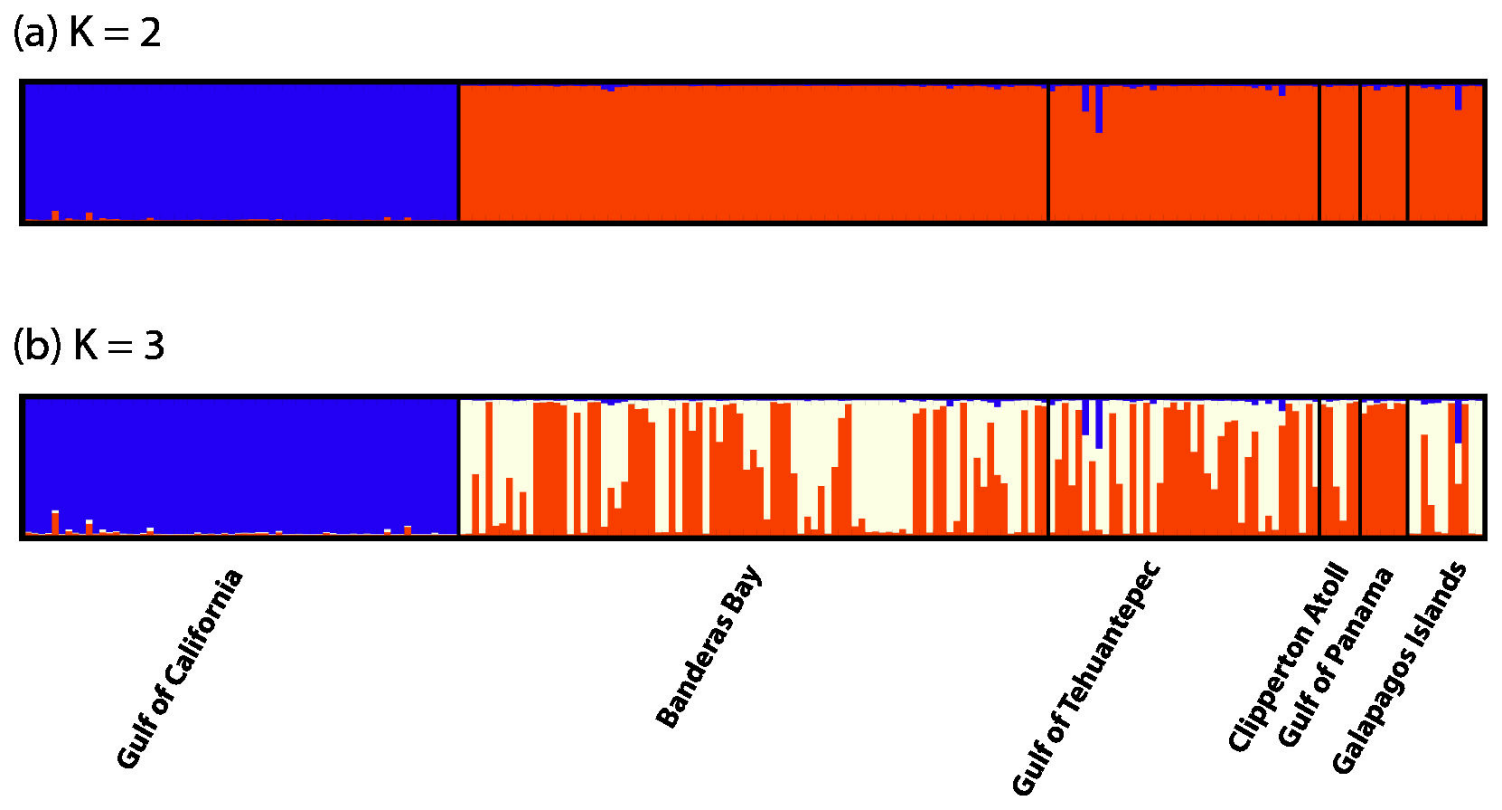
Structure plot of *S. glynni* populations from the tropical and subtropical eastern Pacific. Structure plot of *S. glynni* populations based on allelic frequencies at 11 microsatellite loci. Each bar in the graph represents the probability that a sample belongs to a particular color-coded population. Each graph represents analyses for a particular number of populations (K) run under an admixture with a correlated allele frequency model. (a) Major differentiation occurred between the Gulf of California population and populations in the ETP (*k* = 2). (b) Additional clustering occurred in the ETP, yet did not correspond to location, depth or host morphospecies (*k* = 3).

**Figure 2:**
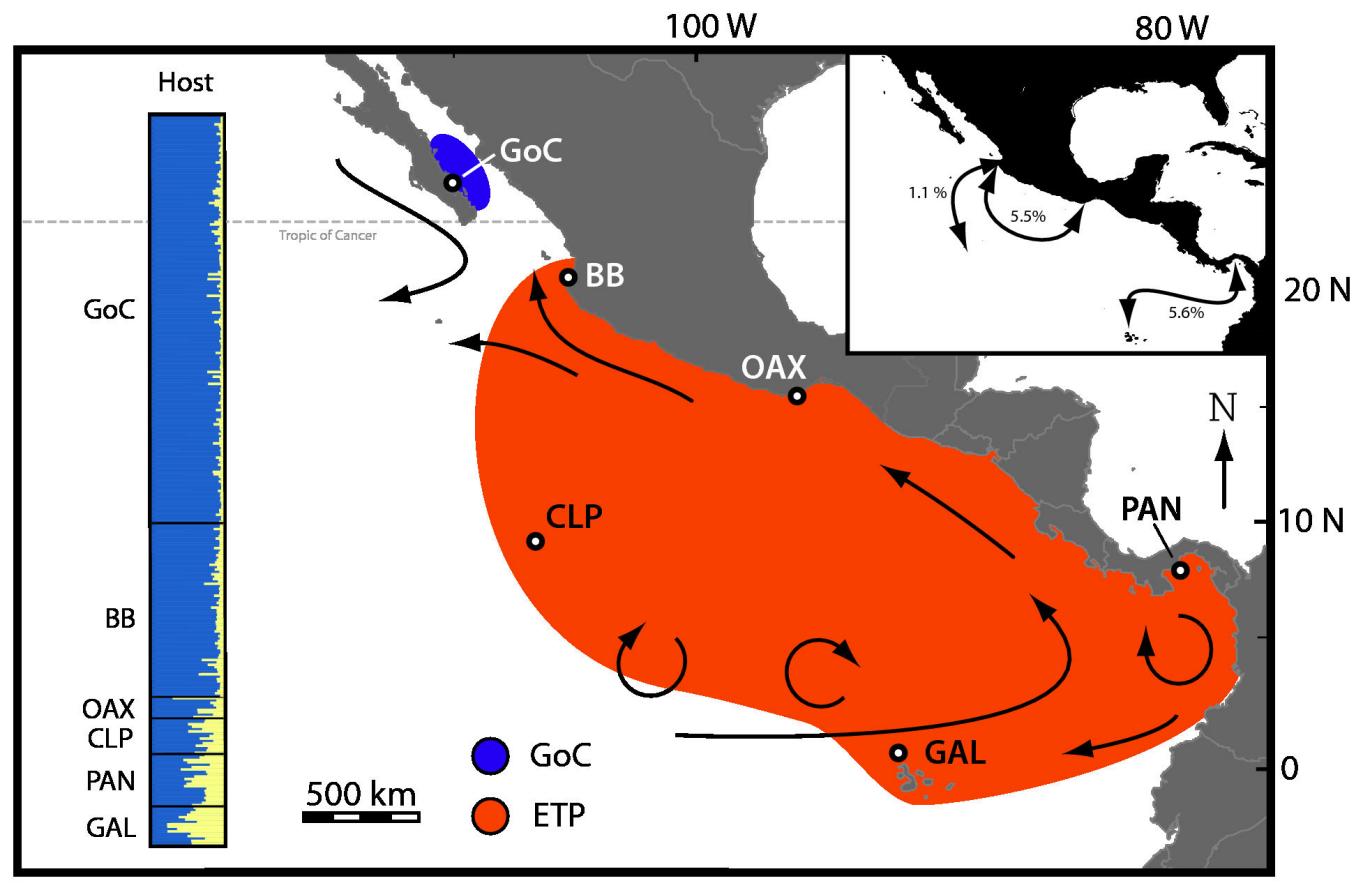
Biogeographic map of the eastern Pacific showing differentiated populations of *S. glynni*. Differentiated populations of *S. glynni* as determined by statistical analyses of microsatellite data. Open circles represent sampling locations with name abbreviations and arrows depict prevailing ocean currents: Gulf of California (GoC), Banderas Bay (BB), Gulf of Tehuantepec (OAX), Clipperton Atoll (CLP), Gulf of Panama (PAN) and Galapagos Islands (GAL). Populations in the subtropical Gulf of California (shaded in purple) were genetically distinct from tropical populations (orange). The left most Structure plot shows genetic homogeneity across *Pocillopora* type 1 populations throughout the eastern Pacific (data obtained from [16]). The inset panel in the upper right quantifies the instances where a cloned *S. glynni* genotype was found between geographically distant locations (proportions = individuals shared/total # unique MLGs between two locations).

Two well supported populations were detected using the Evanno et al. method to process the Structure results (*k* = 2; ln P(D) = -2351.4; Figure 1, Figure S1), and corresponded to the subtropical GoC region and the ETP (Figure 2). The posterior probabilities for increasing values of K continued to rise until *k* = 6, at which they stabilize and/or decline (Figure S1). Further examination of Structure runs at *k* = 3 showed consistency between runs and additional structuring within the ETP region and may represent two cryptic populations within the region (Figure 1; ln P(D) = -2230.4 & Δruns = 1.0; Figure S1); however, this additional subdivision appeared haphazard and does not relate to geographic location or depth. Subsequent analyses on the ETP region only, and using the location prior feature, revealed similar patterns as above with no further subdivision in relation to geographic location in the ETP (data not shown). Additional analyses using BAPS supported the two regional populations (*k* = 2; log(ml) = -2489.7) with no further subdivision of the ETP, even when analyzed in isolation (*k* = 1; log(ml) = -1780.3).

 None of the Structure analyses indicated meaningful population subdivision according to host morphospecies at any location (Figure S2). Analysis of the second order rate of change of K suggested two populations in the GoC and OAX, neither of which corresponded to morphospecies (Figure S2). In one case, four populations were suggested for BB, but these did not align with morphospecies. Examination of the posterior probabilities for each value of K showed similar increases between different K’s up to four and then a large drop in probability between *k* = 4 and 5. Since the method by Evanno et al. considers change in probabilities both before and after a specific K, the analysis in this case is artificially influenced by the large decrease in the posterior probability after *k* = 4 and a single population among morphospecies is the most logical conclusion. Examination of Structure plots for each K supported the above conclusions (Figure S2). Subsequent analyses of these data using BAPS also supported a single population among morphospecies (log(ml) for *k* = 1: GoC, -656.3; BB, -806.1; OAX, -596.0). Lastly, AMOVA’s for each location were not significant at p = 0.0001 (Φ_PT_ = -0.015, GoC; -0.004, BB; -0.007, OAX), as were all pairwise comparisons between morphospecies at each location (p < 0.001, Table S3).

Regional variance analysis based on clusters corresponding to the GoC and ETP yielded highly significant values (p = 0.0001, Table 2) Φ_RT_ = 0.373, Φ_PR_ = 0.040 and Φ_PT_ = 0.398, further validating a tropical-subtropical subdivision. Some significance was observed between populations within the ETP (Φ_PR_), which indicates there may be weak geographical differentiation within the ETP between PAN and both BB and OAX, which was not detected by Structure or BAPS (Table 3). However, the pairwise comparisons of Phi (Φ) values between the GoC population and populations within the ETP showed that differentiation was always greatest between the GoC and all other locations (Table 3).

**Table 2:** Analysis of Molecular Variance (AMOVA) for *S. glynni* in the eastern Pacific from each of six collection locations with regional tropical and subtropical populations (GoC and ETP).

**source**	**df**	**SS**	**MS**	**estimated variance**	**percent of variation**	**fixation index**
**Among Regions**	1	145.39	145.39	1.52	37%	Φ_RT_ = 0.373*
**Among Pops w/in Regions**	4	18.80	4.70	0.10	2%	Φ_PR_ = 0.040*
**Within Pops**	209	513.08	2.45	2.45	60%	Φ_PT_ = 0.398*
**Total**	214	677.27		4.07	100%	

* P < 0.0001

**Table 3:** A pairwise matrix comparing the genetic relationship (Φ_PT_) between all six locations of *S. glynni* in the eastern Pacific.

		**Eastern Tropical Pacific**					
		**Gulf of California**	**Banderas Bay**	**Gulf of Tehuantepec**	**Clipperton Atoll**	**Gulf of Panama**	**Galapagos Islands**
	**Gulf of California**	---					
**Eastern**	**Banderas Bay**	**0.411**	---				
**Tropical**	**Gulf of Tehuantepec**	**0.374**	0.017	---			
**Pacific**	**Clipperton Atoll**	**0.388**	0.018	0.022	---		
	**Gulf of Panama**	**0.379**	**0.146**	**0.124**	0.079	---	
	**Galapagos Islands**	**0.337**	0.045	0.036	0.016	0.129	---

Significant values (sequential Bonferroni corrected P < 0.05) represented in bold.

While the majority of genotypes were restricted to a single sample location, nine MLGs were detected in more than one location (Figure 2, inset). The most geographically widespread of these clones was one found in both PAN and GAL, approximately 1,600 km apart. Seven cloned genotypes were shared between BB and OAX (~ 1,200 km apart) consistent with the predominant surface currents in the region (Figure 2). All 64 MLGs found in the GoC were unique to this location. 

### Environmental data

**Figure 3:**
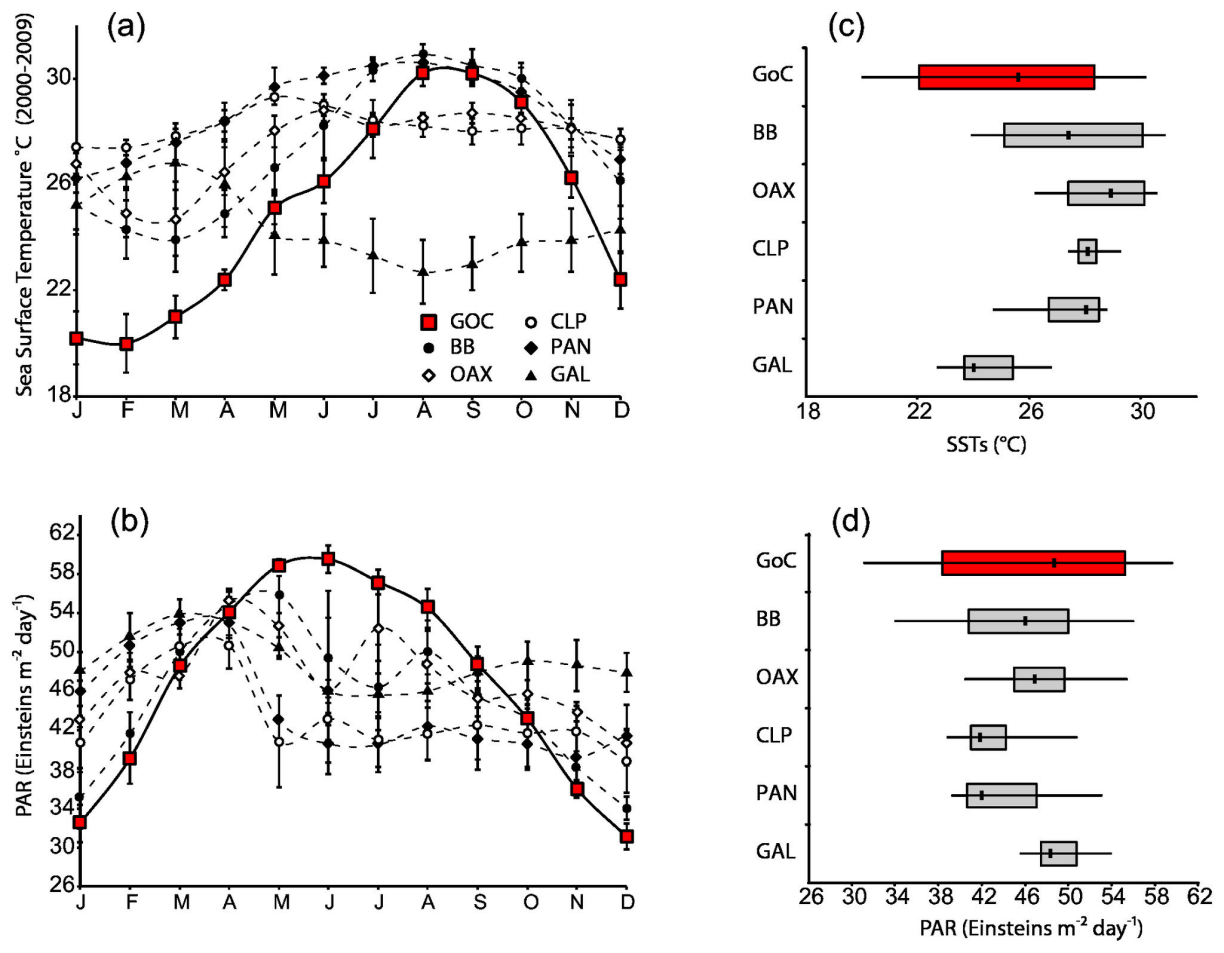
Ten year averages of monthly means of SSTs and PAR for the six sampling locations. Ten year averages of monthly means (2000 - 2009) of sea surface temperatures (SSTs) and photosynthetically available irradiance (PAR) for the six sampling locations. (a) and (c) depict monthly averages and standard deviations at all locations, while (b) and (d) are box plots showing annual mean, first and third quartiles, and maximum and minimum seasonal ranges by location. Monthly averages were acquired from NASA’s Giovanni website.

SSTs and PAR from each location were compared on a monthly and yearly basis from 2000 to 2009 to investigate the relation of environmental conditions with population structuring of *S. glynni* (Figure 3a-d). Sites in the Gulf of California 1) endured the largest yearly fluctuation in monthly averages in both environmental parameters (~10 °C and 28 Einsteins m^-2^ day^-1^, Figure 3c and 3d, respectively); 2) had the coldest monthly average temperature (~20.0 °C SD ± 1.1 in February), at least 4.3 °C colder than any other region (Figure 3a); 3) were exposed to the lowest and highest monthly PAR averages (~31.0 Einsteins m^-2^ day^-1^ SD ± 1.3 in December and ~59.5 Einsteins m^-2^ day^-1^ SD ± 1.4 in June, Figure 3b); and 4) maximal average monthly temperatures (~30.1 °C SD ± 0.5) were only 0.8 °C lower than the highest average among all locations. 

## Discussion

Population genetic approaches offer the resolution necessary to infer key processes in the ecology and evolution of coral-dinoflagellate symbioses. *Symbiodinium glynni* in association with *Pocillopora* type 1 is nearly ubiquitous throughout the eastern Pacific, especially in coastal areas where annual temperatures and water turbidity fluctuate considerably [18]. Stress-tolerant symbioses involving symbionts like *S. glynni* may become increasingly important in the response of coral communities to a warming climate [22], but many uncertainties remain with regard to their potential for dispersal. The patterns observed here and discussed below should redirect speculation on the nature of these associations and highlight the utility of population genetic analyses for elucidating the microevolution of these symbionts.

### Symbiont genotype diversity within and between samples

The routine detection of a single multilocus genotype of *Symbiodinium* per sample makes population genetic studies possible. Among the hundreds of samples analyzed, less than 20% contained detectable genotype mixtures. The extensive sampling of *Pocillopora* type 1 from two study sites in the Gulf of California confirm that colonies are homogeneous for one *S. glynni* genotype over all, or most, of the colony [13], and is consistent with a growing number of microsatellite analyses on *Symbiodinium* in scleractinians and gorgonians that recover a single dominant genotype in 70 to 95% of samples (e.g., *Pseudopterogorgia elisabethae* [10], *Madracis* spp. [11], *Gorgonia ventalina* [26,28], *Montastraea annularis* and *M. faveolata* [27], *Acropora palmata* [45]). In contrast, genotype diversity involving clade C symbionts is reported to be significantly greater per colony [25,29], but this appears to be an artifact of non-specific primer binding, the occurrence of duplicated loci and the presence of a second closely-related symbiont species (unpubl. data; see also [11,12,45]).

*Symbiodinium glynni* genotypes within a colony persist over time, with individual genotypes present in colonies for at least 9 months [13]. These observations support the pioneering work of Goulet and Coffroth [46,47], who detected a single dominant genotype from gorgonian colonies monitored over 10 years. However, data also indicated that a colony could occasionally experience change in the dominant genotype for reasons unknown. The analyses of replicate clonal colonies, or ramets, of *Pocillopora* distributed across a reef demonstrated that the dominant genotype can be replaced in adult colonies over time, but the frequency and rate of this turnover remains undetermined [13]. At spatial scales beyond the colony, *S. glynni* genotypes showed limited spatial partitioning among and between locations separated by several kilometers (present study, [13]). Across the eastern Pacific, genotypic diversity was high and linkage between loci low, suggesting that sexual recombination occurs at a frequency great enough to mask the effect of clonal evolution among *S*. *glynni* strains [10].

### No subdivision observed across host morphospecies

The specificity for a particular host species, genus or family is a common ecological trait among *Symbiodinium* spp. [16,20,32,44,48,49]. Reproductive isolation due to host specialization is presumed to be critical in the process of generating and maintaining *Symbiodinium* diversity [48]. *Symbiodinium glynni* genotypes sorted randomly among the *damicornis, meandrina*, and *verrucosa* morphospecies comprising *Pocillopora* type 1 (*sensu* [16]). The lack of population subdivision for *S. glynni* across morphospecies of type 1 suggests that there are no intracellular differences imparting selective pressure on the evolution of reproductive isolation ([50], Figure S2, Table S3), and adds to the body of evidence indicating that the *Pocillopora* type 1 is a cohesive species despite the distinct morphologies found within this genetic grouping [16].

### Connectivity among tropical *S*. *glynni* populations

No apparent genetic partitioning was found among populations distributed across the tropical region encompassing 1000’s of square kilometers from mainland central Mexico and Clipperton Atoll to the Gulf of Panama and the Galapagos Islands (Figures 1 & 2). Cloned genotypes were routinely observed within and between sites involving spatial scales from 2-100 km indicating *S. glynni* (and *Pocillopora* larvae) frequently disperse over these distances. Surprisingly, identical MLGs were recovered from locations separated by as much as 1,600 km (Figure 2 inset), and suggest that certain clones can successfully disperse over long distances. Many clones may have migrated with their host in a stepping-stone fashion; however, populations of *Pocillopora* are fragmented along the coast of Central America and few to no landmasses exist between offshore islands sites. Therefore, effective dispersal must be possible over distances much larger than previously documented for other marine eukaryotic microbes (e.g., [3,5,6]). 

The dispersal and recruitment of *S. glynni* genotypes must be strongly influenced by *Pocillopora*’s mode of symbiont acquisition, and thus affects genetic structuring. Recent genetic analysis [16] found little population differentiation among *Pocillopora* type 1 across the eastern Pacific (Figure 2), and is consistent with reports of weak genetic structure among populations separated by 500 km or more (e.g., [53,54]). The apparent lack of geographic structuring throughout much of the ETP suggests that the broadcast spawned larvae of *Pocillopora* persist for many days in the water column, similar to laboratory experiments with brooded larvae (e.g., [55]). Aided by the homogenizing action of complex surface currents [14,17,56], spawned *Pocillopora* larvae are probably disbursed throughout the ETP region [57]. *Pocillopora* transmit their symbionts vertically during oogenesis [30,31,58], with fertilized eggs containing over one hundred symbiont cells that divide as larvae begin to develop [58]. Assuming a larva settles in an adequate location, survives and grows, the co-migrating symbiont proliferates as the colony matures. Over time these migrant symbionts can contribute significantly to the local *S. glynni* gene pool. While nothing is known about the longevity of “free-living” *S. glynni* cells, the entrainment of symbionts in the larvae of *Pocillopora* explains in large part the presence of identical MLGs in distant locations and the high genetic connectivity observed for symbiont populations in the ETP (Figure 2). 

The maintenance of gene flow over large distances by *S. glynni* contrasts with assertions that Clade B *Symbiodinium* in the Caribbean exhibit limited gene flow across populations of hosts with horizontal modes of symbiont acquisition [10,26,28]. Assuming all genotypes infect and compete equally well, it has been proposed that the competitive interactions between symbionts for host habitat are density dependent when larvae acquire symbionts from the environment [27]. Host colonies expel millions of viable symbiont cells daily to regulate symbiont densities [51]. Therefore, larvae that settle on a reef are exposed to high environmental concentrations of symbiont genotypes (i.e. clones) that dominate the surrounding host population. In order for a particular horizontally transmitted symbiont clone, or individual, to establish itself in a new region it must disperse passively via water currents and compete with locally dominant clones by successfully infecting and proliferating within the cells of a compatible host [10,52]. Over successive generations the proliferation of common clonal lines among coral recruits will lead to a few MLGs dominating a given reef, significantly effecting genetic structuring over relatively short distances [10,25–28]. In contrast, the vertical transmission of *S. glynni*, combined with its host dispersal, may exclude it from this density dependent competition.

### Local adaptation of and competitive exclusion by subtropical populations of *S*. *glynni*

Compared to the broad latitudinal connectivity observed among host populations, *S. glynni* populations in the subtropical Gulf of California were genetically distinct from all other populations in the Eastern Tropical Pacific (Figure 1 & 2). Opposing currents and abrupt differences in temperature and salinity can restrict dispersal and migration into and out of the Gulf (Figure 2; [59,60]). However, seasonal alterations in the prevailing surface currents allow occasional biological exchange [59] and may explain why *Pocillopora* type 1 shows no indication of population subdivision in this region (Figure 2 [16], but see [61]). What, then, explains the apparent lack of symbiont migrants? The discordant patterns of genetic differentiation between the host and symbiont suggest processes that maintain genetic concordance between host and symbiont populations in tropical locations are different in higher latitude environments.

Environmental conditions that pose no physical barriers to dispersal may explain the strong genetic structure exhibited by the symbiont, while maintaining a confluent host population. Temperature and light gradients are important in the distribution and prevalence of *Symbiodinium* spp. [20,62,63]. The Gulf of California experiences extreme annual fluctuations in temperature (~10°C) and irradiance (~27 Einsteins m^-2^ day^-1^) relative to the other eastern Pacific locations (Figure 3a-d). We propose that genetic differentiation in symbiont populations is maintained by competitive displacement by locally adapted symbiont genotypes [64]. Host genetics indicate that tropical genotypes of *S. glynni* may often disperse into the Gulf carried by their larval hosts. Upon settlement, however, successful “migrant” symbionts are presumably out competed by locally adapted genotypes (and possibly vice versa for subtropical migrants arriving at more tropical locals). Strains (i.e., MLGs) of *S. glynni* from the environment can enter and displace the resident strain in adult *Pocillopora* colonies [13], a process more recently verified in the symbiosis biology of other Pocilloporidae [64]. Therefore, corals that inherit their symbionts maternally may form new associations with compatible symbiont strains, or species, during the life of a colony and explains how host connectivity is maintained, while *S. glynni*’s is not. 

If locally adapted genotypes are migrating into adjacent populations, then it may seem unusual that subtropical and tropical genotypes of *S. glynni* where never found to co-occur within the same colony. However, rapid competition and replacement would likely proceed early in the host’s life, during larval settlement and juvenile growth, and only adult colonies were sampled during the course of this research. Additional sampling of juveniles during the late summer or early fall would assess this possibility. Similarly, sampling near the mouth of the Gulf of California, where environmental conditions transition from subtropical to more tropical, would delineate the geographic boundaries of each population and maximize the chance of finding colonies experiencing competitive displacement. Finally, it is possible that the host microsatellite loci examined for *Pocillopora* type I were not adequately polymorphic and the discordance between host and symbiont populations is the result of markers with low resolution. However, the loci’s high allelic diversity (up to 16 alleles, average/locus = 10.8) and confidence in delimiting individuals (probability of identity = 4.2 x 10^-6^) in a population with high genotypic diversity (R = 0.88) make this possibility unlikely [16]. 

The genetically distinct *S. glynni* genotypes of the subtropics may have evolved recently during a range expansion from the south following the last glaciation (~10-18 KYA). However, the considerable genetic differentiation between tropical and subtropical populations may possibly reflect a longer period of isolation that began during the more extreme conditions associated with the last inter-glacial period. Fossil records indicate that *Pocillopora* were extensive throughout the Gulf of California during the late Pleistocene (~125 KYA; reviewed in [65]), but whether refuge populations endured the last, or preceding, Pleistocene ice ages is unknown. 

While environmental gradients clearly influence the distribution and prevalence of different *Symbiodinium* clades and species [20,62,63], the findings here are among the first empirical data indicating that adaptation to local environmental conditions may be important in driving genetic differentiation and contributing to speciation among symbiotic dinoflagellates. This possibility seems intuitive since hydrological features commonly regulate the genetic structuring of free-living dinoflagellates [5,6] and other marine microalgae [3]. Ultimately, comparative physiology of *Pocillopora* colonies from both populations under controlled laboratory settings and/or reciprocal transplanting is needed to substantiate the relative influence of local adaptation and competition on the genetic structure of *S. glynni*.

### Implications for conservation in the eastern Pacific

The ability of *Pocillopora* type 1 and *S. glynni* to disperse throughout much of the eastern Pacific suggests that the shallow water ecosystems they create may be resilient to local disturbances and capable of recovery by migration of individuals from surrounding sources. However, the lack of gene flow between tropical and subtropical populations of *S. glynni* indicates that these populations may be differently adapted to fluctuating environmental conditions (e.g., in light and temperature). Populations in the Gulf of California are relatively small and may be particularly vulnerable to extinction if host populations were lost to severe environmental degradation. Conversely, as the climate continues to change, symbiont populations adapted to variable environmental conditions might be a source of important physiological diversity. While more information is needed to evaluate the relative exchange of the eastern Pacific with the rest of the Pacific, the broad thermal tolerance of *S. glynni* [18] and its ability to disperse large distances with its host suggest that this region might provide a source of *Pocillopora* whose symbionts are adapted to warmer and more variable environmental conditions. 

## Supporting Information

Figure S1 **Plots derived from the method by Evanno et al.** **2005 using the second order rate of change of the Ln P(D) to determine the appropriate value of K based on runs in Structure**.
a) Plot of the average Ln P(D) (±SD) over five runs for each value of K. b) Result plot of the second order rate of change showing a clear peak at K = 2, representing the genetic break between the GoC and the ETP. c) Raw data from the ten consecutive Structure runs with five replicates at each K and used for the above analyses.
(DOC)

Figure S2 **Structure plots of *S. glynni* populations showing a lack of population subdivision according to *Pocillopora* morphospecies in the GoC (**a**), BB (**b**) and OAX (**c**).** Structure plots are arranged in order of increasing K and the data is ordered according to morphology of the colony from which it was collected.
(EPS)

Table S1 **Description of microsatellite loci used in this study.** T_a_ = annealing temperature, A_e_ = effective alleles with standard error in parenthesis. Subscript numbers following the repeat motif indicate the number of repeats in the initial cloned sequence that was used to develop locus primers.
(DOC)

Table S2 **Haploid allele frequencies and sample size by location for *S. glynni*.**
(DOC)

Table S3 **A pairwise matrix comparing the genetic relationship (Φ_PT_) between *S. glynni* populations based on *Pocillopora* morphospecies in the eastern Pacific.** Analyses were conducted in three different locations: (a) Gulf of California, (b) Banderas Bay and (c) Gulf of Tehuantepec. Significant values (sequential Bonferroni corrected P < 0.05) represented in bold.
(DOC)

Text S1 **Supplemental materials and methods further describing microsatellite analysis and data analysis.**
(DOC)
