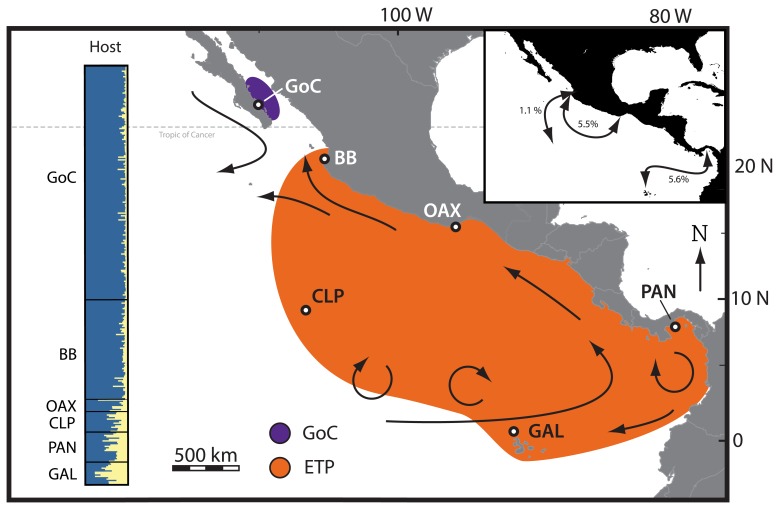# Correction: Long-Range Dispersal and High-Latitude Environments Influence the Population Structure of a “Stress-Tolerant” Dinoflagellate Endosymbiont

**DOI:** 10.1371/annotation/de510c87-50ec-4edb-9691-fd409939d3b1

**Published:** 2014-01-02

**Authors:** D. Tye Pettay, Todd C. LaJeunesse

There were errors introduced during the preparation of this manuscript for publication. The colors represented in Figures 1 and 2 are incorrect. Please view the correct Figure 1 here 

**Figure:**
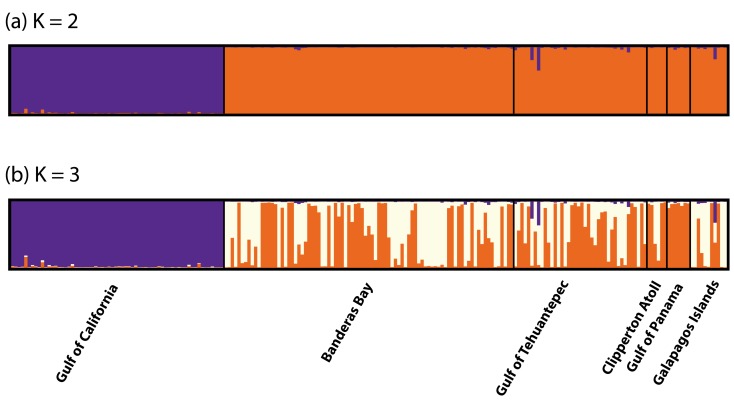


and the correct Figure 2 here 

**Figure:**