# Supplementary material for: Long-Range Dispersal and High-Latitude Environments Influence the Population Structure of a “Stress-Tolerant” Dinoflagellate Endosymbiont
Source: PLoS One. 2013 Nov 5;8(11):e79208. doi: 10.1371/journal.pone.0079208 (PMC3818422; doi:10.1371/journal.pone.0079208)
Supplement: Figure S1 — Plots derived from the method by Evanno et al. 2005 using the second order rate of change of the Ln P(D) to determine the appropriate value of K based on runs in Structure. a) Plot of the average Ln P(D) (±SD) over five runs for each value of K. b) Result plot of the second order rate of change showing a clear peak at K = 2, representing the genetic break between the GoC and the ETP. c) Raw data from the ten consecutive Structure runs with five replicates at each K and used for the above analyses. (DOC) [file pone.0079208.s001.doc]

a) Ln P(D) = L(K)

b) ∆K

K lnP(D) = L(K)

1 -3019.1

1 -3019.5

1 -3019.4

1 -3019.5

1 -3019.2

2 -2351.6

2 -2351.4

2 -2351.5

2 -2351.4

2 -2351.3

3 -2230.7

3 -2231.4

3 -2230.9

3 -2230.4

3 -2230.5

4 -2201.2

4 -2200.8

4 -2201.4

4 -2092.1

4 -2201.3

5 -2175.5

5 -2169.5

5 -2174.5

5 -2176.8

5 -2171.4

6 -2305

6 -2246

6 -2217.6

6 -2286.1

6 -2244.7

7 -2233.5

7 -2222.8

7 -2052.8

7 -2395.2

7 -2090.1

8 -2071.3

8 -2066

8 -2149.1

8 -3888.8

8 -2081.1

9 -2200.1

9 -3642.8

9 -2087.5

9 -2276.1

9 -2074.2

10 -2163.5

10 -2417.5

10 -2124.7

10 -2095.8

10 -2138.5

Figure S1. Plots derived from the method by Evanno *et al.* 2005 using the second order rate of change of the Ln P(D) to determine the appropriate value of K based on runs in Structure. a) Plot of the average Ln P(D) (±SD) over five runs for each value of K. b) Result plot of the second order rate of change showing a clear peak at K = 2, representing the genetic break between the GoC and the ETP. c) Raw data from the ten consecutive Structure runs with five replicates at each K and used for the above analyses.
