# Supplementary material for: Long-Range Dispersal and High-Latitude Environments Influence the Population Structure of a “Stress-Tolerant” Dinoflagellate Endosymbiont
Source: PLoS One. 2013 Nov 5;8(11):e79208. doi: 10.1371/journal.pone.0079208 (PMC3818422; doi:10.1371/journal.pone.0079208)
Supplement: Table S1 — Description of microsatellite loci used in this study. Ta = annealing temperature, Ae = effective alleles with standard error in parenthesis. Subscript numbers following the repeat motif indicate the number of repeats in the initial cloned sequence that was used to develop locus primers. (DOC) [file pone.0079208.s003.doc]

Table S1. Description of microsatellite loci used in this study. T_a_ = annealing temperature, A_e_ = effective alleles with standard error in parenthesis. Subscript numbers following the repeat motif indicate the number of repeats in the initial cloned sequence that was used to develop locus primers.

| **locus** | **primer sequence (5' - 3')** | **repeat motif** | **T_a_ (°C)** | **size range (bp)** | **number of alleles** | **A_e_** |
| --- | --- | --- | --- | --- | --- | --- |
| D1Sym9 | F - CAGAAGCCCAATTATATGCGGCA (FAM) | (GTT)_6_ | 57 | 106 - 115 | 4 | 1.14 (0.09) |
|  | R - AGGATGATGAGCATGCCGACG |  |  |  |  |  |
| D1Sym11 | F - TGAAATCTCACTCAGAGTCGGAC (FAM) | (AC)_13_ | 57 | 151 - 161 | 6 | 2.42 (030) |
|  | R - GCAGACAGTGATTTCAGTTCCGA |  |  |  |  |  |
| D1Sym14 | F - TCTCAGTGGAAAGCATTGTGG (FAM) | (CT)_11_ AT (CT)_4_ | 55 | 173 - 185 | 7 | 1.57 (0.33) |
|  | R - TCGTCTGAATCAGGATCTGACG |  |  |  |  |  |
| D1Sym17 | F - TGTGAATGCTTCTTGGGGTG (HEX) | (CA)_8_ | 57 | 143 - 167 | 13 | 4.36 (0.78) |
|  | R - TCATGCTTGTCCGTGAGCAG |  |  |  |  |  |
| D1Sym34 | F - ACCTGAGACCTGAGTGTTGC (FAM) | (CAAA)_9_ CACA (CAAA)_4_ (GAAACAAA)_2_ (CAAA)_13_ | 55 | 332 - 428 | 29 | 6.22 (1.15) |
|  | R - ATCATGGGCAGAGCTCCTGG |  |  |  |  |  |
| D1Sym67 | F - GAATCCAGATGGTGCCTGC (VIC) | (ATC)_8_ | 57 | 131 - 149 | 7 | 2.40 (0.37) |
|  | R - CAAAGGTAGCCGATTGTCTC |  |  |  |  |  |
| D1Sym77a | F - CCACTGAGATTGGTAGGTGAA (PET) | (TTC)_5_ CT (CTTCCT)_2_ C (TTC)_4_ | 55 | 169 - 184 | 6 | 2.03 (0.35) |
|  | R - ACCGATGGTGTTTGTGACTCG |  |  |  |  |  |
| D1Sym77b | F - CCACTGAGATTGGTAGGTGAA (PET) | (TTC)_5_ CT (CTTCCT)_2_ C (TTC)_4_ | 55 | 184 - 193 | 4 | 1.32 (0.14) |
|  | R - ACCGATGGTGTTTGTGACTCG |  |  |  |  |  |
| D1Sym87 | F - CCTATGACTCCAAGGGTGACG (FAM) | (GAAG)_7_ | 57 | 244 - 268 | 7 | 2.91 (0.48) |
|  | R - AGACATACCTCGGTCTTGTC |  |  |  |  |  |
| D1Sym88 | F - TTGTCAGACTGAATGCTCCA (NED) | (CTTT)_3_ G (TTTC)_7_ (TCTCTTTC)_2_ TCTTTTT (CT)_3_ (TCTT)_2_ (TTTC)_2_ T (CTT)_4_ | 55 | 227 - 235 | 3 | 1.02 (0.02) |
|  | R - GTGTTCAAGCGACATCCCA |  |  |  |  |
| D1Sym92 | F - GCGTTTGACACAAGGATCCCT (FAM) | (CCTA)_6_ (CCTG)_3_ | 57 | 124 - 132 | 3 | 1.07 (0.03) |
|  | R - TTGGGATGCTCTTGGCGAC |  |  |  |  |  |
