# Supplementary material for: Long-Range Dispersal and High-Latitude Environments Influence the Population Structure of a “Stress-Tolerant” Dinoflagellate Endosymbiont
Source: PLoS One. 2013 Nov 5;8(11):e79208. doi: 10.1371/journal.pone.0079208 (PMC3818422; doi:10.1371/journal.pone.0079208)
Supplement: Table S2 — Haploid allele frequencies and sample size by location for S. glynni. (DOC) [file pone.0079208.s004.doc]

Table S2. Haploid allele frequencies and sample size by location for *S. glynni.*

| **Locus** | **Allele** | **GoC** | **BB** | **OAX** | **CLP** | **PAN** | **GAL** |
| --- | --- | --- | --- | --- | --- | --- | --- |
| **D1Sym9** | **N** | 73 | 98 | 48 | 8 | 8 | 18 |
|  | **106** | 0.000 | 0.020 | 0.000 | 0.000 | 0.000 | 0.000 |
|  | **109** | 1.000 | 0.847 | 0.792 | 1.000 | 1.000 | 1.000 |
|  | **112** | 0.000 | 0.102 | 0.208 | 0.000 | 0.000 | 0.000 |
|  | **115** | 0.000 | 0.031 | 0.000 | 0.000 | 0.000 | 0.000 |
| **D1Sym11** | **N** | 73 | 92 | 45 | 8 | 8 | 16 |
|  | **151** | 0.219 | 0.065 | 0.000 | 0.375 | 0.375 | 0.125 |
|  | **153** | 0.438 | 0.739 | 0.644 | 0.375 | 0.625 | 0.500 |
|  | **155** | 0.055 | 0.141 | 0.133 | 0.000 | 0.000 | 0.188 |
|  | **157** | 0.233 | 0.043 | 0.111 | 0.125 | 0.000 | 0.188 |
|  | **159** | 0.041 | 0.011 | 0.089 | 0.125 | 0.000 | 0.000 |
|  | **161** | 0.014 | 0.000 | 0.022 | 0.000 | 0.000 | 0.000 |
| **D1Sym14** | **N** | 73 | 98 | 48 | 8 | 8 | 16 |
|  | **173** | 0.014 | 0.000 | 0.000 | 0.000 | 0.000 | 0.000 |
|  | **175** | 0.000 | 0.010 | 0.021 | 0.000 | 0.000 | 0.000 |
|  | **177** | 0.000 | 0.949 | 0.813 | 0.875 | 0.375 | 1.000 |
|  | **179** | 0.301 | 0.041 | 0.167 | 0.000 | 0.625 | 0.000 |
|  | **181** | 0.274 | 0.000 | 0.000 | 0.125 | 0.000 | 0.000 |
|  | **183** | 0.397 | 0.000 | 0.000 | 0.000 | 0.000 | 0.000 |
|  | **185** | 0.014 | 0.000 | 0.000 | 0.000 | 0.000 | 0.000 |
| **D1Sym17** | **N** | 73 | 93 | 48 | 8 | 8 | 18 |
|  | **143** | 0.014 | 0.000 | 0.000 | 0.000 | 0.000 | 0.000 |
|  | **145** | 0.479 | 0.011 | 0.000 | 0.000 | 0.000 | 0.000 |
|  | **147** | 0.247 | 0.011 | 0.063 | 0.000 | 0.000 | 0.000 |
|  | **149** | 0.260 | 0.140 | 0.083 | 0.000 | 0.125 | 0.167 |
|  | **151** | 0.000 | 0.086 | 0.333 | 0.000 | 0.000 | 0.000 |
|  | **153** | 0.000 | 0.194 | 0.333 | 0.250 | 0.375 | 0.389 |
|  | **155** | 0.000 | 0.172 | 0.125 | 0.375 | 0.375 | 0.056 |
|  | **157** | 0.000 | 0.108 | 0.021 | 0.125 | 0.125 | 0.000 |
|  | **159** | 0.000 | 0.054 | 0.000 | 0.000 | 0.000 | 0.111 |
|  | **161** | 0.000 | 0.097 | 0.021 | 0.125 | 0.000 | 0.167 |
|  | **163** | 0.000 | 0.086 | 0.000 | 0.125 | 0.000 | 0.056 |
|  | **165** | 0.000 | 0.022 | 0.021 | 0.000 | 0.000 | 0.000 |
|  | **167** | 0.000 | 0.022 | 0.000 | 0.000 | 0.000 | 0.056 |
| **D1Sym34** | **N** | 73 | 98 | 47 | 8 | 8 | 18 |
|  | **332** | 0.000 | 0.010 | 0.000 | 0.000 | 0.000 | 0.000 |
|  | **336** | 0.000 | 0.010 | 0.000 | 0.000 | 0.000 | 0.000 |
|  | **340** | 0.000 | 0.051 | 0.000 | 0.000 | 0.000 | 0.000 |
|  | **344** | 0.000 | 0.173 | 0.021 | 0.000 | 0.000 | 0.000 |
|  | **348** | 0.000 | 0.020 | 0.000 | 0.000 | 0.000 | 0.000 |
|  | **356** | 0.000 | 0.010 | 0.000 | 0.000 | 0.000 | 0.000 |
|  | **370** | 0.000 | 0.031 | 0.000 | 0.000 | 0.000 | 0.000 |
|  | **372** | 0.014 | 0.000 | 0.000 | 0.000 | 0.000 | 0.000 |
|  | **374** | 0.000 | 0.010 | 0.000 | 0.000 | 0.000 | 0.056 |
|  | **376** | 0.027 | 0.000 | 0.000 | 0.000 | 0.000 | 0.000 |
|  | **378** | 0.014 | 0.010 | 0.000 | 0.000 | 0.000 | 0.000 |
|  | **380** | 0.055 | 0.020 | 0.000 | 0.000 | 0.000 | 0.000 |
|  | **384** | 0.082 | 0.000 | 0.043 | 0.000 | 0.000 | 0.000 |
|  | **388** | 0.055 | 0.010 | 0.021 | 0.000 | 0.000 | 0.000 |
|  | **390** | 0.000 | 0.041 | 0.021 | 0.000 | 0.000 | 0.000 |
|  | **392** | 0.137 | 0.000 | 0.000 | 0.000 | 0.000 | 0.000 |
|  | **394** | 0.000 | 0.061 | 0.000 | 0.000 | 0.000 | 0.000 |
|  | **396** | 0.301 | 0.082 | 0.043 | 0.000 | 0.500 | 0.167 |
|  | **400** | 0.247 | 0.020 | 0.170 | 0.000 | 0.125 | 0.056 |
|  | **402** | 0.000 | 0.000 | 0.000 | 0.000 | 0.000 | 0.167 |
|  | **404** | 0.068 | 0.122 | 0.191 | 0.375 | 0.000 | 0.056 |
|  | **408** | 0.000 | 0.153 | 0.170 | 0.250 | 0.375 | 0.222 |
|  | **410** | 0.000 | 0.010 | 0.000 | 0.000 | 0.000 | 0.000 |
|  | **412** | 0.000 | 0.051 | 0.213 | 0.125 | 0.000 | 0.167 |
|  | **414** | 0.000 | 0.010 | 0.000 | 0.000 | 0.000 | 0.000 |
|  | **416** | 0.000 | 0.010 | 0.085 | 0.125 | 0.000 | 0.056 |
|  | **418** | 0.000 | 0.000 | 0.000 | 0.125 | 0.000 | 0.000 |
|  | **420** | 0.000 | 0.041 | 0.021 | 0.000 | 0.000 | 0.056 |
|  | **424** | 0.000 | 0.031 | 0.000 | 0.000 | 0.000 | 0.000 |
|  | **428** | 0.000 | 0.010 | 0.000 | 0.000 | 0.000 | 0.000 |
| **D1Sym67** | **N** | 73 | 98 | 48 | 8 | 8 | 18 |
|  | **131** | 0.014 | 0.010 | 0.000 | 0.250 | 0.000 | 0.000 |
|  | **134** | 0.740 | 0.000 | 0.021 | 0.000 | 0.000 | 0.000 |
|  | **137** | 0.082 | 0.082 | 0.000 | 0.000 | 0.000 | 0.000 |
|  | **140** | 0.164 | 0.327 | 0.354 | 0.500 | 1.000 | 0.278 |
|  | **143** | 0.000 | 0.449 | 0.542 | 0.250 | 0.000 | 0.333 |
|  | **146** | 0.000 | 0.112 | 0.063 | 0.000 | 0.000 | 0.167 |
|  | **149** | 0.000 | 0.020 | 0.021 | 0.000 | 0.000 | 0.222 |
| **D1Sym77** | **N** | 62 | 98 | 48 | 7 | 8 | 16 |
|  | **169** | 0.000 | 0.041 | 0.188 | 0.000 | 0.000 | 0.063 |
|  | **172** | 0.000 | 0.010 | 0.000 | 0.143 | 0.000 | 0.000 |
|  | **175** | 0.000 | 0.082 | 0.083 | 0.000 | 0.000 | 0.313 |
|  | **178** | 0.032 | 0.765 | 0.667 | 0.857 | 0.625 | 0.375 |
|  | **181** | 0.952 | 0.092 | 0.063 | 0.000 | 0.250 | 0.250 |
|  | **184** | 0.016 | 0.010 | 0.000 | 0.000 | 0.125 | 0.000 |
| **D1Sym77b** | **N** | 62 | 98 | 48 | 7 | 8 | 16 |
|  | **178** | 0.032 | 0.061 | 0.021 | 0.000 | 0.000 | 0.000 |
|  | **181** | 0.290 | 0.000 | 0.000 | 0.000 | 0.000 | 0.000 |
|  | **184** | 0.000 | 0.020 | 0.000 | 0.000 | 0.000 | 0.000 |
|  | **187** | 0.016 | 0.857 | 0.729 | 1.000 | 1.000 | 0.938 |
|  | **190** | 0.403 | 0.061 | 0.250 | 0.000 | 0.000 | 0.063 |
|  | **193** | 0.258 | 0.000 | 0.000 | 0.000 | 0.000 | 0.000 |
| **D1Sym87** | **N** | 73 | 98 | 45 | 8 | 8 | 18 |
|  | **244** | 0.356 | 0.010 | 0.133 | 0.000 | 0.000 | 0.000 |
|  | **248** | 0.137 | 0.184 | 0.156 | 0.000 | 0.000 | 0.278 |
|  | **252** | 0.164 | 0.531 | 0.533 | 0.625 | 0.375 | 0.333 |
|  | **256** | 0.041 | 0.204 | 0.156 | 0.375 | 0.625 | 0.056 |
|  | **260** | 0.096 | 0.020 | 0.000 | 0.000 | 0.000 | 0.278 |
|  | **264** | 0.178 | 0.051 | 0.000 | 0.000 | 0.000 | 0.000 |
|  | **268** | 0.027 | 0.000 | 0.022 | 0.000 | 0.000 | 0.056 |
| **D1Sym88** | **N** | 73 | 98 | 48 | 8 | 8 | 18 |
|  | **227** | 0.055 | 0.000 | 0.000 | 0.000 | 0.000 | 0.000 |
|  | **231** | 0.000 | 1.000 | 1.000 | 1.000 | 1.000 | 1.000 |
|  | **235** | 0.945 | 0.000 | 0.000 | 0.000 | 0.000 | 0.000 |
| **D1Sym92** | **N** | 73 | 98 | 48 | 8 | 8 | 18 |
|  | **124** | 0.027 | 0.000 | 0.000 | 0.000 | 0.000 | 0.056 |
|  | **128** | 0.973 | 0.969 | 0.979 | 1.000 | 1.000 | 0.944 |
|  | **132** | 0.000 | 0.031 | 0.021 | 0.000 | 0.000 | 0.000 |
