# Supplementary material for: Long-Range Dispersal and High-Latitude Environments Influence the Population Structure of a “Stress-Tolerant” Dinoflagellate Endosymbiont
Source: PLoS One. 2013 Nov 5;8(11):e79208. doi: 10.1371/journal.pone.0079208 (PMC3818422; doi:10.1371/journal.pone.0079208)
Supplement: Table S3 — A pairwise matrix comparing the genetic relationship (ΦPT) between S. glynni populations based on Pocillopora morphospecies in the eastern Pacific. Analyses were conducted in three different locations: (a) Gulf of California, (b) Banderas Bay and (c) Gulf of Tehuantepec. Significant values (sequential Bonferroni corrected P < 0.05) represented in bold. (DOC) [file pone.0079208.s005.doc]

Table S3. A pairwise matrix comparing the genetic relationship (Φ_PT_) between *S. glynni* populations based on *Pocillopora* morphospecies in the eastern Pacific. Analyses were conducted in three different locations: (a) Gulf of California, (b) Banderas Bay and (c) Gulf of Tehuantepec. Significant values (sequential Bonferroni corrected P < 0.05) represented in bold.

(a)

| **Morphospecies** | ***P. capitata*** | ***P. damincornis*** | ***P. meandrina*** | ***P. verrucosa*** |
| --- | --- | --- | --- | --- |
| ***P. capitata*** | --- |  |  |  |
| ***P. damincornis*** | 0.000 | --- |  |  |
| ***P. meandrina*** | 0.000 | 0.006 | --- |  |
| ***P. verrucosa*** | 0.000 | 0.000 | 0.000 | --- |

(b)

| **Morphospecies** | ***P. capitata*** | ***P. damincornis*** | ***P. meandrina*** | ***P. verrucosa*** |
| --- | --- | --- | --- | --- |
| ***P. capitata*** | --- |  |  |  |
| ***P. damincornis*** | 0.000 | --- |  |  |
| ***P. meandrina*** | 0.000 | 0.000 | --- |  |
| ***P. verrucosa*** | 0.026 | 0.000 | 0.046 | --- |

(c)

| **Morphospecies** | ***P. capitata*** | ***P. damincornis*** | ***P. eydouxi*** | ***P. meandrina*** | ***P. verrucosa*** |
| --- | --- | --- | --- | --- | --- |
| ***P. capitata*** | --- |  |  |  |  |
| ***P. damincornis*** | 0.000 | --- |  |  |  |
| ***P. eydouxi*** | 0.000 | 0.000 | --- |  |  |
| ***P. meandrina*** | 0.000 | 0.000 | 0.038 | --- |  |
| ***P. verrucosa*** | 0.027 | 0.000 | 0.050 | 0.000 | --- |
