# Supplementary material for: Long-Range Dispersal and High-Latitude Environments Influence the Population Structure of a “Stress-Tolerant” Dinoflagellate Endosymbiont
Source: PLoS One. 2013 Nov 5;8(11):e79208. doi: 10.1371/journal.pone.0079208 (PMC3818422; doi:10.1371/journal.pone.0079208)
Supplement: Text S1 — Supplemental materials and methods further describing microsatellite analysis and data analysis. (DOC) [file pone.0079208.s006.doc]

**Supplemental Materials & Methods**

*Microsatellite Analysis*

Scoring D1Sym77

Primers for locus D1Sym77 consistently amplified two independent alleles. The presence of two alleles at this locus appears to be from a partial chromosome duplication event early in the evolution of this symbiont lineage [29]. Detailed analysis of allelic data throughout the sampling range has revealed two distributions of alleles that are distinct and non-overlapping, and easily binned into low (77a) and high-sized (77b) fragments with two alleles from a single size distribution never co-occurring within an individual in ninety-nine percent of the samples. Because alleles were relatively independent of each other, each was scored as a separate locus with the smaller allele sized designated as one locus (77a) and the larger alleles as a second locus (77b).

Scoring Multiple MLG’s in single samples

Most microsatellite analyses to date have found a single MLG per colony (see discussion), however work on clade C *Symbiodinium* have suggested the presence of multiple MLGs in a majority of the colonies sampled [30, 34]. Dinoflagellate genomes contain regions of highly repetitive DNA that are readily apparent during microsatellite development [15, 16, 43, 44] and these contrasting observations (one dominant genotype vs. multiple genotypes in a sample) are more likely due to non-specific binding and/or loci that have undergone recent duplication, rather than high genotype diversity within individual colonies [unpubl. data; see also 45, 15, 16, 44]. Therefore, multiple alleles at a particular locus were scored when a second clear peak was found in the expected size range and was at least one-third the size of the dominant peak. The presence of multiple peaks was interpreted to indicate that a sample contained more than one haploid genotype. The occasional exclusion of small ambiguous peaks may occasionally cause a low background genotype to be missed, however this conservative approach prevents the overestimation of genetic diversity [46]. Additionally, while this approach may slightly skew the results of diversity on a per colony basis, it should have little impact on the population structure analyses since these mixed genotype samples did not result in additional allelic diversity.

*Data Analysis*

Clonality

Populations of organisms that commonly reproduce asexually will tend to have a high frequency of clones. Therefore statistical calculations based on allele frequencies can be negatively biased [47]. For example, linkage analysis on a population that is highly clonal may indicate that loci are in linkage disequilibrium (LD), when they may not. Similarly, AMOVA’s based on genetic distance measurements between pairs of samples are potentially wrongly influenced by high numbers of duplicated MLGs in a data set. Therefore, a population with high clonality often appears genetically different from other populations not dominated by a particular clone. For these reasons, all analyses (unless stated otherwise) were conducted with duplicated MLGs removed at each sampling location.

Summary Statistics

The probability of identity (PI = (Σp_i_^2^)^2^ - Σp_i_^4^, where p_i_ is the frequency of the *i*-th allele at a locus) was calculated to determine the power to resolve genetically distinct individuals. The PI is an estimate of the probability that two unrelated individuals drawn at random will by chance have the same MLG [48]. Individual PI’s are calculated for each locus and an overall PI for all loci is the product of each individual locus PI. PI values between 0.01 – 0.0001 are believed to be reasonably low enough for population studies, with values lower than 0.01 adequate for mark-recapture studies on population size estimation [49]. Haploid genetic diversity (h = 1 - Σp_i_^2^) gives an indication that two individuals drawn at random will be genetically different, while information index (I) is a measure of allelic diversity [48]. Lastly, clonal richness (R) which is equal to (G – 1)/(N – 1), where G = # of unique MLG’s and N = total sample size, was also calculated to give the frequency of unique genotypes and an indication of the contribution of asexual reproduction.

Using Structure on Haploid Data

The software Structure (Version 2.3.2) was used to overcome biases of assigning populations by location by using the microsatellite data to cluster MLG based on their genetic similarities irrespective of sample origin. Briefly, this software uses a Bayesian clustering approach to probabilistically assign individuals to populations [51]. The model assumes there are K populations characterized by a set of allele frequencies, with the assumptions of unlinked loci that are in linkage and Hardy-Weinberg equilibrium (HWE) within populations [51]. While the assumption of HWE does not directly apply to haploid organisms, as long as the organism recombines, which *Symbiodinium* does, and there is not a large signal of LD between loci the ploidy is irrelevant.  *Symbiodinium* does reproduce asexually and removing repetitive MLGs prevents the biasing of Structure analyses due to clonality. Concerning HWE specifically, this theory poses that in an idealized theoretical population allele frequencies remain constant from generation to generation. In the case of haploid organisms, the maintenance of constant allele frequencies (e.g., HWE) can still be assumed for neutral loci with a balance between factors such as mutation, selection, migration and drift, and that violation of this assumption (e.g., nonrandom mating, strictly asexual reproduction) will yield populations that are in linkage disequilibrium.

BAPS

BAPS utilizes a different algorithm that relies mostly on a greedy stochastic optimization procedure, only using a Markov chain Monte Carlo (MCMC) procedure for complex data sets [58, 59]. This procedure allows for faster analyses, yet typically yields results similar to Structure for known population structuring [59]. Mixture analyses were run in BAPS for both the geographic and morphospecies clustering, using the “cluster by groups” option, K ranging from one to ten (geographic) or one to eight (morphospecies) and five replicates for each K. The K with the highest log marginal likelihood value was considered the appropriate number of clusters.

**References**

15. Pettay D., LaJeunesse T. 2007 Microsatellites from clade B *Symbiodinium* spp. specialized for Caribbean corals in the genus *Madracis*. *Molecular Ecology Notes* **7**(6), 1271-1274.

16. Pettay D.T., LaJeunesse T.C. 2009 Microsatellite loci for assessing genetic diversity, dispersal and clonality of coral symbionts in ‘stress-tolerant’ clade D *Symbiodinium*. *Molecular Ecology Resources* **9**(3), 1022-1025. (doi:10.1111/j.1755-0998.2009.02561.x).

29. Wham D.C., Pettay D.T., LaJeunesse T.C. 2011 Microsatellite loci for the host-generalist “zooxanthella” *Symbiodinium trenchi* and other Clade D *Symbiodinium*. *Conservation Genetic Resources*, 1-4. (doi:10.1007/s12686-011-9399-2).

30. Howells E., Van Oppen M., Willis B. 2009 High genetic differentiation and cross-shelf patterns of genetic diversity among Great Barrier Reef populations of *Symbiodinium*. *Coral Reefs* **28**(1), 215-225.

34. Magalon H., Baudry E., Husté A., Adjeroud M., Veuille M. 2006 High genetic diversity of the symbiotic dinoflagellates in the coral *Pocillopora meandrina* from the South Pacific. *Marine Biology* **148**(5), 913-922.

43. Jaeckisch N., Yang I., Wohlrab S., Glockner G., Kroymann J., Volgel H., Cembella A., John U. 2011 Comparative genomic and transcriptomic characterization of the toxic marine dinoflagellate *Alexandrium ostenfeldii*. *PloSone*, e28012.

44. Pinzón J.H., Devlin-Durante M.K., Weber M., Baums I.B., LaJeunesse T.C. 2011 Microsatellite loci for *Symbiodinium A3* (*S. fitti*) a common algal symbiont among Caribbean *Acropora* (stony corals) and Indo-Pacific giant clams (*Tridacna*). *Conservation Genetics Resources* **3**, 45-47.

45. Santos S., Coffroth M. 2003 Molecular genetic evidence that dinoflagellates belonging to the genus *Symbiodinium* Freudenthal are haploid. *The Biological Bulletin* **204**(1), 10-20.

46. Anderson T., Haubold B., Williams J.T., Estrada-Franco J.G., Richardson L., Mollinedo R., Bockarie M., Mokili J., Mharakurwa S., French N., et al. 2000 Microsatellite markers reveal a spectrum of population structures in the malaria parasite *Plasmodium falciparum*. *Molecular Biology and Evolution* **17**(10), 1467-1482.

47. Arnaud-Haond S., Duarte C.M., Alberto F., Serrão E.A. 2007 Standardizing methods to address clonality in population studies. *Molecular Ecology* **16**(24), 5115-5139. (doi:10.1111/j.1365-294X.2007.03535.x).

48. Peakall R., Smouse P.E. 2006 GENALEX 6: genetic analysis in Excel. Population genetic software for teaching and research. *Molecular Ecology Notes* **6**, 288-295.

49. Waits L., Luikart G., Taberlet P. 2001 Estimating the probability of identity among genotypes in natural populations: cautions and guidelines. *Molecular Ecology* **10**(1), 249-256.

51. Pritchard J., Stephens M., Donnelly P. 2000 Inference of population structure using multilocus genotype data. *Genetics* **155**(2), 945.

58. Corander J., Marttinen P., Mäntyniemi S. 2006 A Bayesian method for identification of stock mixtures from molecular marker data. *Fishery Bulletin* **104**(4), 550-558.

59. Latch E.K., Dharmarajan G., Glaubitz J.C., Rhodes O.E. 2006 Relative performance of Bayesian clustering software for inferring population substructure and individual assignment at low levels of population differentiation. *Conservation Genetics* **7**(2), 295-302. (doi:10.1007/s10592-005-9098-1).
